# Modulation of Cancer Cell Autophagic Responses by Graphene-Based Nanomaterials: Molecular Mechanisms and Therapeutic Implications

**DOI:** 10.3390/cancers13164145

**Published:** 2021-08-18

**Authors:** Biljana Ristic, Ljubica Harhaji-Trajkovic, Mihajlo Bosnjak, Ivana Dakic, Srdjan Mijatovic, Vladimir Trajkovic

**Affiliations:** 1 Institute of Microbiology and Immunology, Faculty of Medicine, University of Belgrade, 11000 Belgrade, Serbia; biljana.ristic@med.bg.ac.rs (B.R.); ivana.dakic@med.bg.ac.rs (I.D.); 2 Department of Neurophysiology, Institute for Biological Research “Sinisa Stankovic”, National Institute of Republic of Serbia, University of Belgrade, 11060 Belgrade, Serbia; ljubica.harhaji@ibiss.bg.ac.rs; 3 Institute of Histology and Embryology, Faculty of Medicine, University of Belgrade, 11000 Belgrade, Serbia; mihajlo.bosnjak@med.bg.ac.rs; 4 Clinic for Emergency Surgery, Clinical Centre of Serbia, 11000 Belgrade, Serbia; srdjan.mijatovic@med.bg.ac.rs

**Keywords:** graphene, nanomaterial, cancer, autophagy, apoptosis, necrosis

## Abstract

**Simple Summary:**

Graphene-based nanomaterials (GNM) are one-to-several carbon atom-thick flakes of graphite with at least one lateral dimension <100 nm. The unique electronic structure, high surface-to-volume ratio, and relatively low toxicity make GNM potentially useful in cancer treatment. GNM such as graphene, graphene oxide, graphene quantum dots, and graphene nanofibers are able to induce autophagy in cancer cells. During autophagy the cell digests its own components in organelles called lysosomes, which can either kill cancer cells or promote their survival, as well as influence the immune response against the tumor. However, a deeper understanding of GNM-autophagy interaction at the mechanistic and functional level is needed before these findings could be exploited to increase GNM effectiveness as cancer therapeutics and drug delivery systems. In this review, we analyze molecular mechanisms of GNM-mediated autophagy modulation and its possible implications for the use of GNM in cancer therapy.

**Abstract:**

Graphene-based nanomaterials (GNM) are plausible candidates for cancer therapeutics and drug delivery systems. Pure graphene and graphene oxide nanoparticles, as well as graphene quantum dots and graphene nanofibers, were all able to trigger autophagy in cancer cells through both transcriptional and post-transcriptional mechanisms involving oxidative/endoplasmic reticulum stress, AMP-activated protein kinase, mechanistic target of rapamycin, mitogen-activated protein kinase, and Toll-like receptor signaling. This was often coupled with lysosomal dysfunction and subsequent blockade of autophagic flux, which additionally increased the accumulation of autophagy mediators that participated in apoptotic, necrotic, or necroptotic death of cancer cells and influenced the immune response against the tumor. In this review, we analyze molecular mechanisms and structure–activity relationships of GNM-mediated autophagy modulation, its consequences for cancer cell survival/death and anti-tumor immune response, and the possible implications for the use of GNM in cancer therapy.

## 1. Introduction

**Figure 1:**
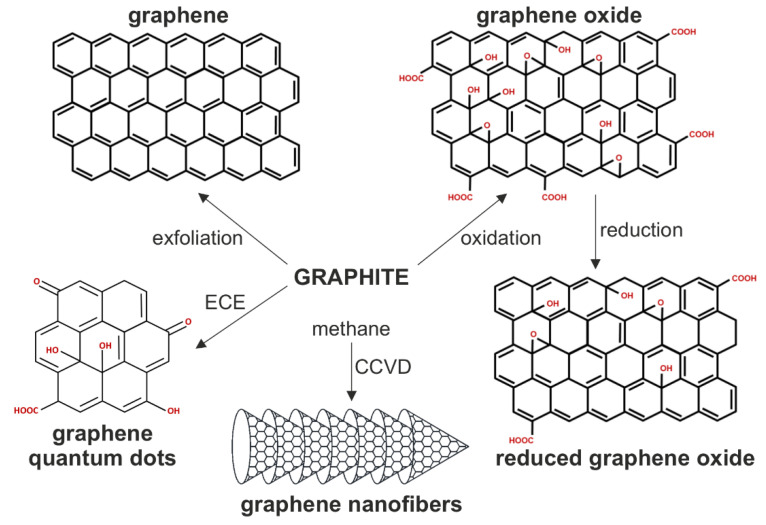
The structure of biologically relevant GNM. The chemical structure of GNM and the main methods for their synthesis are presented. CCVD, catalytic chemical vapor deposition; ECE; electrochemical exfoliation.

Graphene is a single layer of sp^2^-hybridized carbon atoms in a honeycomb structure, produced by top-down exfoliation of graphite or various bottom-up synthetic approaches [1]. Graphene-based nanomaterials (GNM) include nanodimensional (<100 nm in at least one dimension) flakes or ribbons of pure graphene, graphene oxide (GO), and reduced GO (rGO), as well as graphene quantum dots (GQD), all consisting of single to several graphene layers (Figure 1). GO is a hydrophilic derivative of graphene with carboxyl, hydroxyl, and epoxide groups, produced by oxidation of graphite or bottom-up synthesis from a carbon source such as glucose, while rGO is an intermediate structure between the pure graphene and highly oxidized GO [2,3]. GQD are up to 100 nm-wide oval photoluminescent sheets of graphene that might contain heteroatoms such as oxygen and hydrogen, produced by electrochemical exfoliation of graphite or bottom-up synthesis from various carbon sources [4]. There are also graphene nanofibers (GNF; also known as carbon nanofibers), cylindrical nanostructures with graphene layers arranged as stacked cones, cups, or plates, produced from a carbon source such as methane by chemical vapor deposition in the presence of a transition metal catalyst [5,6]. The unique electronic structure, high surface-to-volume ratio, and relatively low toxicity make GNM potentially useful in nanomedicine as bioimaging agents, biosensors, drug-delivery systems, tissue-engineering scaffolds, and anticancer therapeutics [7]. GNM absorb light and kill cancer cells via photothermal or photodynamic effects [8,9]. Graphene, GO, and rGO nanoparticles convert near-infrared (NIR) light into heat for cancer cell ablation [10,11,12], while photoexcited GQD transforms molecular oxygen into highly cytotoxic reactive oxygen species (ROS) [13]. Non-excited GNM also display cytotoxic activity towards various types of tumor cells [14].

Cellular internalization of various nanoparticles, including GNM, triggers macroautophagy (hereafter autophagy), an evolutionarily conserved homeostatic mechanism for lysosomal degradation of cytoplasmic components [15,16,17]. Autophagy acts as a recycling pathway in the absence of nutrients or as a quality-control pathway to eliminate dysfunctional/misfolded proteins and aged or damaged organelles [18]. It proceeds through sequential steps, including engulfment of cytoplasmic content by double-membrane vesicles known as autophagosomes, their fusion with lysosomes, and degradation of autophagic cargo in autolysosomes [18]. Autophagy suppresses genome instability and carcinogenesis by preventing the accumulation of damaged proteins/organelles, oxidative stress, and chronic tissue inflammation, as well as by serving as a backup cell death program in apoptosis-deficient cancer cells [19,20]. Moreover, autophagy induced by some chemotherapeutics can contribute to apoptotic/necrotic tumor cell demise or function as an alternative programmed cell death pathway [20]. On the other hand, established tumors might exploit autophagy as a survival mechanism to combat metabolic, hypoxic, oxidative, or drug-induced stress, thus providing a rationale for clinical trials using autophagy inhibitors to increase cancer sensitivity to chemotherapy or radiation [21].

**Figure 2:**
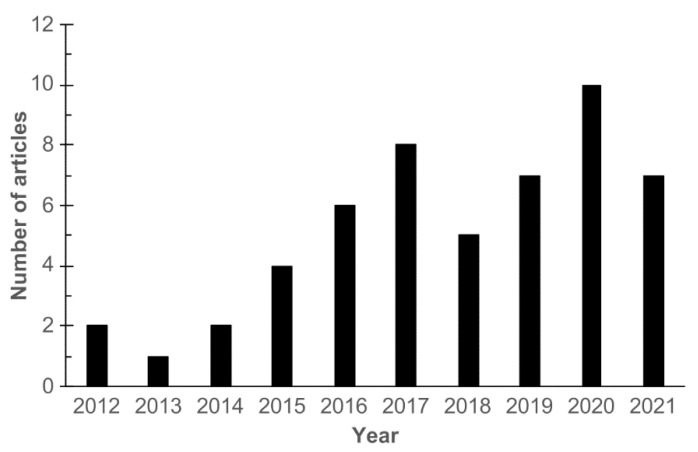
The number of research articles on autophagy modulation by GNM. The relevant articles were identified by searching the PubMed database using the search terms “graphene” and “autophagy”.

The number of experimental studies on autophagic responses of mammalian cells to GNM has been steadily increasing since 2012 (Figure 2). A review from 2017 analyzed the signaling pathways involved in autophagy induction by GNM [22]. However, a number of reports has been published since then, providing novel insights into the molecular mechanisms of GNM-mediated autophagy induction. Moreover, the role of autophagy in GNM anticancer action and the underlying structure–activity relationships, to the best of our knowledge, have not been systematically evaluated thus far. In this review, we analyze autophagy modulation by GNM in cancer cells, focusing on its molecular mechanisms, structure–activity requirements, consequences for cancer cell survival/death, and the possible implications for the use of GNM in cancer therapy.

## 2. Autophagy Regulation and Role in Cell Death

**Figure 3:**
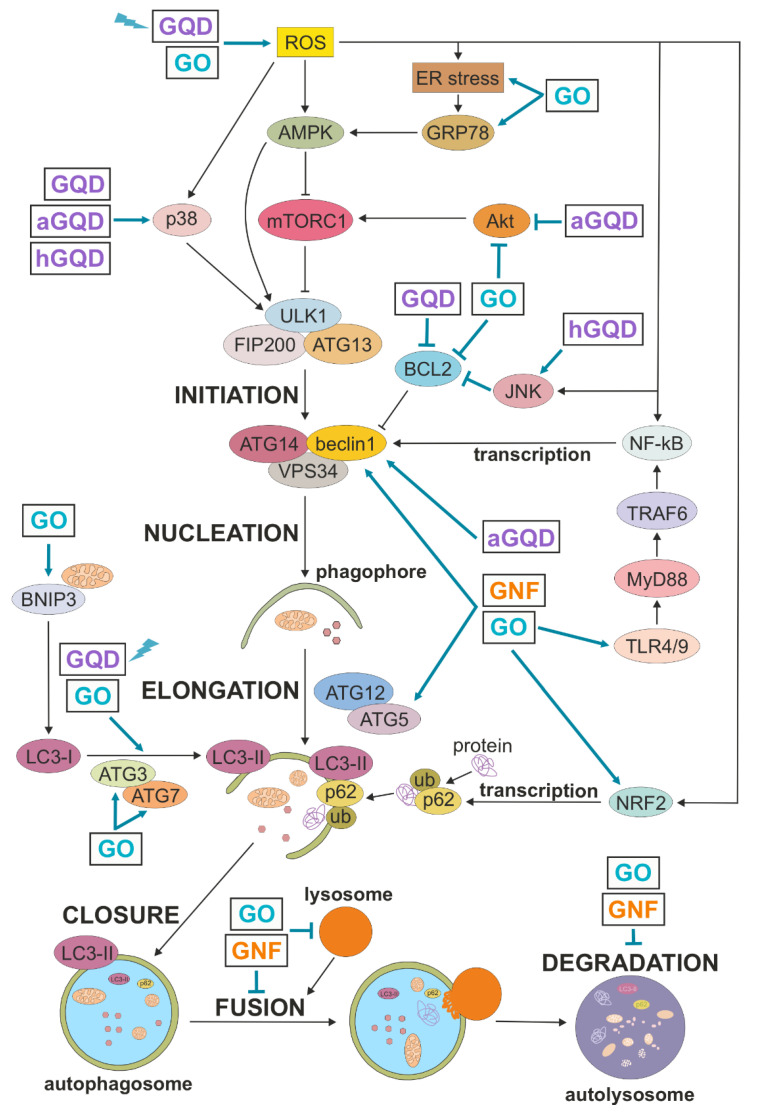
Autophagy regulation and its modulation by GNM. A simplified map of autophagy regulation is presented, including the sites of action of GNM shown as arrow-headed (activation/increase) or bar-headed lines (inhibition/decrease). GQD and GO are shown to directly modulate LC3 conversion because no specific underlying mechanism is described. The presented interactions are described in more detail in the main text. aGQD, amino-GQD; AMPK, AMP-activated protein kinase; ATG, autophagy-related; BCL2, B cell lymphoma 2; BNIP3, BCL2-interacting protein 3; ER, endoplasmic reticulum; FIP200, focal adhesion kinase family interacting protein of 200 kD; GNF, graphene nanofibers; GO, graphene oxide; GQD, graphene quantum dots; GRP78, glucose-regulated protein 78; hGQD, hydroxy-GQD; JNK, c-Jun N-terminal kinase; LC3, microtubule-associated light chain 3; NF-κB, nuclear factor-κB; NRF2, nuclear factor erythroid 2-related factor 2; mTORC1, mechanistic target of rapamycin complex 1; MyD88, myeloid differentiation factor 88; ROS, reactive oxygen species; TLR, Toll-like receptor; TRAF6, TNF receptor associated factor 6; ub, ubiquitin; ULK1, Unc-51-like kinase 1; VPS34, vacuolar protein sorting 34; lightning symbol denotes photoexcitation.

Autophagic machinery is set in motion by integrating various stress (e.g., oxidative, metabolic, cytotoxic, endoplasmic reticulum stress) signals by different protein kinases and transcription factors, including AMP-activated protein kinase (AMPK), protein kinase B/AKT, mechanistic target of rapamycin (mTOR), mitogen-activated protein kinases (MAPK), nuclear factor-κB (NF-κB), and nuclear factor erythroid 2-related factor 2 (NRF2), as schematically presented in Figure 3 (out of many signaling molecules and transcription factors involved in autophagy regulation, these were selected based on their relevance for autophagy modulation by GNM). Autophagy initiation is followed by transcription of autophagy-related (ATG) genes and highly orchestrated sequential activation of post-translationally modified ATG proteins organized in functional complexes, leading to autophagosome nucleation, elongation, closure, and ultimately, fusion with the lysosome [23]. Briefly, autophagosome biogenesis is initiated by recruiting the complex of Unc-51-like kinase 1 (ULK1, mammalian ATG1 homolog), ATG13, and focal adhesion kinase family interacting protein of 200 kD (FIP200) to the phagophore assembly site. Vesicle nucleation continues with ULK1-mediated activation of the complex containing beclin-1 (mammalian ATG6 homolog), ATG14, and class III phosphoinositide 3 kinase (PI3K) vacuolar protein sorting 34 (VPS34), which generates phosphatidylinositol 3-phosphate as a platform for further ATG recruitment. The next step, vesicle elongation, is mediated by the two ubiquitin-like conjugation systems: first, ATG5 is conjugated to ATG12, which then enables ATG3/ATG7-mediated conjugation of phosphatidylethanolamine to microtubule-associated light chain 3 (LC3)-I, a cytoplasmic form of ATG8 homolog LC3. This leads to generation and autophagosome localization of LC3-II, which promotes the expansion of the autophagosomal membrane and its closure and fusion with the lysosome, where the ubiquitinated cytoplasmic material bound to autophagic cargo receptors such as sequestosome 1 (p62) is ultimately degraded.

Autophagy regulates cancer cell death in a complex, context-dependent manner, displaying either cytotoxic or cytoprotective actions determined by the cell type, autophagy trigger, its duration, and/or extent [19,20,21]. In general, autophagy is a pro-survival stress response that removes damaged and potentially harmful organelles (such as ROS-producing mitochondria) or provides basic building blocks for cell metabolism by recycling non-essential cellular components during nutrient/energy deprivation [18]. Accordingly, deficient or blocked autophagy can be detrimental to the cells due to an impaired quality control/removal of harmful cellular material. On the other hand, prolonged overactivation of the autophagy-lysosomal pathway can cause autophagic cell death or promote apoptosis, necrosis, and necroptosis through selective degradation of organelles (e.g., mitochondria) and pro-survival cellular proteins, or extensive bulk self-digestion beyond the point allowing cell survival [24]. Autophagic cell death mechanistically depends on the autophagic machinery and is characterized by extensive cytoplasmic vacuolization and lysosomal degradation in the absence of nuclear condensation and cell membrane damage as markers of apoptosis and necrosis, respectively. However, some members of autophagic machinery might independently of autophagy intersect with various cell death programs, which adds a further layer of complexity to autophagy-cell death interaction [24].

The recent guidelines recommend that due to its complexity, autophagy should be analyzed at several levels and using different methods [25]. For example, the expression of ATG genes is examined by reverse transcription-PCR, the level, activation, and interactions of ATG proteins are assessed by immunoblotting, while autophagosomes and autolysosomes can be visualized by transmission electron microscopy (TEM) or fluorescence microscopy. One of the most frequently used methods for autophagy analysis is the immunoblot measurement of the conversion of cytoplasmic LC3-I to its lipidated, autophagosome-localized form LC3-II [25]. However, the fact that many ATG proteins have autophagy-independent functions and may not even be essential for canonical autophagy [26] makes it difficult to decipher autophagy status by judging the ATG expression alone. Moreover, autophagosomes are degraded during autophagy, thus the increase in the accumulation of autophagosomes and associated molecules, including LC3-II, may also result from their reduced degradation upon autophagy inhibition [25]. In that context, it is essential to assess autophagic flux, a measure of autophagic degradation activity that reflects the dynamic nature of autophagy. The useful approaches for autophagic flux estimation include LC3-II analysis in the presence of lysosomal inhibitors that block its degradation, and the measurement of p62, the cargo receptor degraded in autolysosomes together with ubiquitinated cytoplasmic material [25]. The role of autophagy in cell death could be assessed by using pharmacological inhibitors of autophagosome formation (e.g., class III PI3K inhibitors) or lysosomal degradation (e.g., chloroquine or bafilomycin A1). However, since these agents can modulate cell survival/death independently of autophagy [27,28], genetic inactivation of crucial autophagy mediators (e.g., beclin-1, ATG5/7, LC3) is recommended for this purpose.

## 3. Autophagy Modulation by GNM in Cancer Cells

While GNM interaction with the autophagy network mostly resulted in an increase in LC3 conversion and/or autophagic flux, a blockade of autophagic flux was observed in some studies, as summarized in Table 1 and Figure 3, and described in more detail below.

### 3.1. Autophagy Induction by GNM

The increase in LC3-I/LC3-II conversion and/or LC3 aggregation in various cancer cell types exposed to GO nanoparticles [29,30,31,32,33,34,35,36,37,38] and non-excited/photoexcited GQD [39,40,41] indicates an accumulation of autophagosomes (Table 1). Accordingly, ultrastructural TEM analysis of cancer cells treated with GNM confirmed the presence of double-membrane autophagosomes and/or single-membrane autolysosomes containing cytoplasmic material [33,34,35,36,37,38,39,40,42]. The accompanying decrease in p62 levels and/or effective colocalization of autophagosomes and lysosomes in GNM-treated cancer cells is consistent with the augmented autophagic turnover [30,31,32,33,34,35,39]. Accordingly, GO-mediated increase in autophagic flux was directly confirmed by enhanced accumulation of LC3-II in cancer cells in which its degradation was blocked by lysosomal inhibitors [29,35]. It should be noted that in some experiments, no decrease in p62 levels was observed [35,40], possibly because it was not involved in the delivery of autophagic cargo or because its autophagic degradation was counterbalanced by transcriptional upregulation. Importantly, administration of GO nanoparticles to mice with colon cancer increased the expression of LC3 in the tumor tissue, indicating the ability of GNM to induce autophagy in vivo [32,33].

### 3.2. Suppression of Autophagic Flux by GNM

Conjugation of GO nanosheets with the lysosomal inhibitor chloroquine efficiently inhibited autophagic flux in lung carcinoma cells [43]. Even in the absence of chloroquine, the accumulation of autophagosomes in cancer cells treated with GO and GNF in some studies was apparently due to a block of the degradative stage of autophagy, rather than to an increase in the formation of autophagic structures [44,45,46,47]. This was confirmed by no further increase in LC3-I/II conversion or LC3 punctuation in the presence of lysosomal inhibitors, intracellular accumulation of the selective autophagy target p62, and/or impaired lysosome function and fusion with autophagosomes, implying a decrease in autophagic turnover [44,45,46,47]. However, p62 is transcriptionally upregulated as a part of autophagy transcriptional program [23], and the increase in p62 mRNA levels in GO-exposed lung carcinoma cells indicates that GNM-mediated accumulation of p62 was at least partly due to the increase in its expression [45]. Indeed, the increase in beclin-1 and ATG5 [44,45], which are crucial for canonical autophagosome formation [26], suggests that GNM did not merely suppress basal autophagy but actually enhanced autophagosome biogenesis while blocking their lysosomal degradation. These findings raise concerns that the autophagy-blocking effect of GNM might have been overlooked in the studies that did not directly assess autophagic flux. On the other hand, tumor cell type-specific effects and the differences in GNM physico–chemical properties and experimental conditions could also contribute to the observed discrepancies. To shed some light on this important issue, we later discuss in more detail the structure–activity relationship underlying the autophagy-blocking capacity of GNM.

## 4. Mechanisms of Autophagy Induction by GNM

Autophagy is regulated both transcriptionally, through activation of various transcription factors and subsequent expression of ATG genes, and post-transcriptionally, by phosphorylation, ubiquitination, and/or acetylation of ATG proteins, which alter their functional activity, structure, and affinity for binding partners [23]. These events are controlled by oxidative stress, endoplasmic reticulum (ER) stress, and several signaling pathways involving AMPK, AKT, mTOR, MAPK, and Toll-like receptors (TLR) [48]. The available data indicate that GNM might trigger an autophagic response in cancer cells by signaling through TLR receptors, as well as by inducing the production of ROS and/or ER stress, which then activate ATG transcription and/or post-transcriptional modifications via AMPK/AKT/mTOR or MAPK signaling. Below we discuss the mechanisms of GNM-mediated modulation of autophagy in cancer cells, which are summed up in Table 1 and schematically depicted in Figure 3.

### 4.1. Transcriptional Induction of Autophagy

Several lines of evidence support the transcriptional upregulation of tumor cell autophagy by GNM. The treatment of lung carcinoma, colon carcinoma, neuroblastoma, and monocyte/macrophage leukemia cells with GO or GQD nanoparticles increased the intracellular levels of beclin-1 [31,33,36,37,40,41,45], a mammalian ortholog of ATG6 required for localization of autophagic proteins to a pre-autophagosomal structure [49]. GO- and GQD-mediated increase in beclin-1 protein levels was associated with the activation and nuclear translocation of its main transcription factor NF-κB [33,36,41] and increase in beclin-1 mRNA [45], indicating transcriptional upregulation of beclin-1 by GNM. Moreover, NF-κB was required for GQD-triggered autophagy, as revealed by the anti-autophagic effect of its pharmacological blockade [41]. On the other hand, the expression of beclin-1 antagonist B-cell lymphoma 2 (BCL2) was inhibited by GO, GQD, and GNF [29,41,42,44,45], thus presumably additionally increasing the availability of beclin-1 for autophagy induction. The expression of LC3 was elevated in GO-exposed nasopharingeal carcinoma cells, and its transcriptional activation was indicated by an increase in LC3 mRNA levels [38]. In addition, both LC3 and p62 mRNA were upregulated in GNF-treated lung carcinoma cells [45]. In osteosarcoma, lung carcinoma, and neuroblastoma cells, GO and GNF increased the protein levels of ATG3, ATG5, and/or ATG7 [29,43,44,45], which are required for lipidation of ATG8 family proteins, including LC3, and their subsequent association with the membrane of autophagic vesicles [50]. The treatment of neuroblastoma cells with GO nanoribbons enhanced the expression of BCL2-interacting protein 3 [37], a transcriptionally regulated pro-apoptotic factor involved in autophagic removal of dysfunctional mitochondria [51]. While these data strongly indicate the ability of GNM to transcriptionally activate autophagy in cancer cells, this remains to be directly confirmed by appropriate transcription assays.

### 4.2. AMPK/AKT/mTOR Signaling

The initiation of autophagy is controlled by the master regulator of cellular metabolism, mTOR-containing mTOR complex 1 (mTORC1), which inhibits autophagy by phosphorylating Unc-51-like autophagy activating kinase 1 (ULK1, mammalian ATG1) [52]. Activation of mTORC1 by growth factor/hormone-regulated kinase AKT suppresses, while inhibition of mTORC1 by intracellular energy sensor AMP-activated protein kinase (AMPK) induces autophagy [52]. Autophagy activation in lung carcinoma cells exposed to amino-functionalized GQD coincided with the inhibition of AKT [40], while the treatment of pheochromocytoma and glioma cells with GO suppressed both AKT and mTOR [44,46]. Pharmacological activation of AKT or mTOR partly prevented GO-mediated LC3-I/LC3-II conversion in pheochromocytoma cells, confirming the role of AKT/mTORC1 signaling axis in GO-induced autophagy [44]. GO-triggered autophagic response in neuroblastoma cells was associated with the activation of AMPK and inhibition of mTOR [31]. Moreover, pharmacological blockade of AMPK prevented GO nanoparticle-induced mTOR suppression, beclin-1 increase, and autophagy induction, suggesting involvement of the AMPK/mTORC1 pathway in GO-mediated beclin-1 expression and autophagy activation [31]. It has been proposed that nanoparticles during cellular internalization might affect the recruitment/activation of cell membrane-localized AKT, thus altering its capacity to activate mTORC1, while internalized nanoparticles could additionally affect activation of mTORC1 by preventing its recruitment to the lysosomal membrane [53]. These possibilities are worthy of further investigation, particularly when having in mind that GNM were readily internalized into tumor cells [29,33,35,36,37,39], possibly via clathrin- and/or caveolae-mediated endocytosis [35,54], and that their internalization was required for autophagy induction [35,41].

### 4.3. MAPK Signaling

MAPK family members extracellular signal-regulated kinase (ERK), c-Jun N-terminal kinase (JNK), and p38 MAPK regulate both the initiation and maturation stages of autophagy [55]. ERK inhibits mTORC1 [56], p38 MAPK directly activates ULK1 [57], while JNK is able to relieve beclin-1 from BCL2-mediated suppression [58]. The LC3 conversion in GO-exposed pheochromocytoma and cervical carcinoma cells was accompanied by phosphorylation of ERK and reduced by its inhibition, indicating an involvement of this MAPK member in GO-triggered autophagic response [35]. On the other hand, GQD-mediated autophagy induction in monocytic leukemia cells was associated with p38 MAPK activation and partly prevented by its pharmacological suppression [41]. Similarly, the suppression of AKT, increase in beclin-1, and subsequent induction of autophagy in lung carcinoma cells treated with amino-GQD were accompanied by activation of p38 MAPK, while no increase in ERK or JNK phosphorylation was observed [40]. Although hydroxylated GQD were somewhat less effective in autophagy induction, they were markedly more potent than their amino-functionalized counterparts in activating p38 MAPK and JNK [40]. Finally, only carboxylated GQD were able to activate ERK, but failed to induce autophagy [40]. The apparent absence of a correlation between MAPK activation and autophagy induction indicates a limited involvement of MAPK in GQD-mediated autophagy in lung carcinoma cells. However, an additional analysis using appropriate pharmacological inhibitors and/or genetic inactivation of specific MAPK family members is required to examine their role in GNM-triggered autophagic response in tumor cells.

### 4.4. TLR Signaling

TLR recognizes pathogen-associated molecular patterns and are crucial components of innate immunity. They are also expressed in a variety of immune and non-immune cell tumors, regulating their proliferation and survival [59]. It has been reported that GO nanoparticles trigger autophagy in colon carcinoma and macrophage leukemia cells through TLR4/TLR9-dependent activation of myeloid differentiation factor 88 (MyD88) and TNF receptor-associated factor 6 (TRAF6), initiating the subsequent nuclear translocation of NF-κB [33,36]. Accordingly, genetic knockdown of TLR4 or TLR9 markedly reduced GO-induced beclin-1 expression and autophagosome-lysosome fusion in cancer cells [33,36]. The authors proposed that autophagy induction by GO nanoparticles was preceded by their endocytosis, which was initiated by binding to membrane-bound TLR4 and further enhanced through subsequent interaction with endosome-localized TLR9. This resembles xenophagy, a form of selective autophagy induced by TLR and other signals, in which cytosolic or vacuolar pathogens are delivered to autophagosomes for destruction [60]. In the latter case, pathogen-containing endosomes interact with autophagosomes to form so-called amphisomes, which eventually fuse with lysosomes [60]. This analogy is supported by other studies in which GQD, GO, and GNF were visualized by TEM in endosome- and/or autophagy-like vesicles within the cancer cells [34,35,37,39,45].

### 4.5. Oxidative Stress

ROS play an important role in autophagy induction by modulating AMPK/mTORC1 and MAPK signaling, as well as ATG transcription and enzymatic activity [48]. Therefore, it is plausible that ROS were upstream mediators responsible for triggering autophagy signaling pathways in GNM-treated cancer cells. Indeed, the treatment with GO, GNF, or both photo-excited and non-excited GQD caused an increase in the intracellular levels of ROS in neuroblastoma, glioma, monocytic leukemia, lung carcinoma, and osteosarcoma cells [29,37,39,41,42,43,45,47]. This was accompanied by the activation and nuclear translocation of redox-sensitive transcription factors NF-κB and NRF2 [29,41,47], which control the expression of beclin-1 and p62, respectively [61,62]. Moreover, pharmacological blockade of NF-κB inhibited GQD-induced autophagy [41], indicating its dependence on ROS-mediated NF-κB activation. There is a question of the source of intracellular ROS generated upon exposure to GNM. In the case of photoexcited GQD, at least some of the ROS production could be attributed to singlet oxygen or superoxide anion generated by GQD themselves through energy or electron transfer to molecular oxygen [39]. On the other hand, as non-excited GNM do not generate ROS, it is conceivable that, in the absence of overt photoexcitation, ROS were produced upon the interaction of GNM with cellular enzymes and/or organelles such as the mitochondria and lysosomes. Accordingly, NADPH oxidase and nitric oxide (NO) synthase were involved in GQD-mediated ROS production in monocytic leukemia cells [41], while GO/GNF-induced oxidative stress in neuroblastoma and lung carcinoma cells coincided with mitochondrial membrane depolarization [37,42,45], which increases ROS generation by the electron transfer chain [63]. Besides mitochondria, lysosomes are an important source of ROS [64], and it is expected that GNM would eventually end up in lysosomes, either via the autophagic or endosomal pathway. Interestingly, non-excited GQD in certain conditions might even act as efficient quenchers of intracellular ROS [65], which might seem to contradict their pro-autophagic capacity. However, our unpublished data demonstrate that GQD. despite their antioxidant effect, were still able to upregulate autophagy in neuroblastoma cells in which oxidative/nitrosative stress was induced by a chemical donor of NO. Moreover, this pro-autophagic action could not be mimicked by classical antioxidants, indicating that, at least in some conditions, GNM might trigger autophagy completely independently of ROS modulation.

### 4.6. ER Stress

Disturbances in ER function can lead to the accumulation of unfolded or misfolded proteins, a condition referred to as ER stress. ER stress activates the unfolded protein response (UPR), which reduces unfolded protein load to maintain cell viability and function, partly by inducing autophagy [66]. For example, it has been reported that glucose-regulated protein 78 (GRP78), an ER protein and a master regulator of UPR with chaperone and calcium-binding properties [67], can increase autophagy in cancer cells through AMPK activation [68]. Interestingly, GO-mediated induction of autophagy in nasopharyngeal carcinoma cells was associated with the increase in GRP78 levels [38]. Moreover, pharmacological blockade of ER stress reduced GO-triggered increase in LC3 transcription and aggregation, indicating a role for ER stress/UPR in the observed autophagic response [38]. As both oxidative stress and AMPK/mTORC1 signaling interact with ER stress in a bidirectional manner [69], it would be interesting to examine the ROS-AMPK/mTORC1-ER stress interplay in GNM-mediated autophagy induction in cancer cells.

## 5. Mechanisms of Autophagic Flux Suppression by GNM

The presence of GNM within autophagosomes, and subsequently autolysosomes, might also be related to their ability to impair the degradative capacity of the latter, resulting in the blockade of autophagic flux [44,45,46,47]. Somewhat unexpectedly, this property was associated with a profound downregulation of autophagy-suppressing AKT/mTORC1 pathway in GO- or GNF-treated pheochromocytoma, lung carcinoma, and glioma cells [44,45,46]. The apparent discrepancy could be explained by the fact that, although inactivated during autophagy initiation, mTORC1 is later reactivated to limit excessive autophagy through negative feedback, as well as to promote its completion via lysosome reformation [70]. Therefore, it is possible that after the initial induction of autophagy, prolonged mTORC1 inhibition by GNM could eventually impair lysosome reformation, thus reducing lysosomal number/size and, subsequently, autophagic flux. While consistent with this hypothesis, the observed reduction of the lysosomal acidic compartment and subsequent decrease in cathepsin and acid phosphatase activity [44,45,46] could also result from alkalization of lysosomes and/or permeabilization of their membrane. Indeed, the latter possibility was supported by rapid translocation of lysosomal permeabilization marker galectin-3 to leaky lysosomes in GO-exposed pheochromocytoma cells [44], as well as by the increased levels of lysosomal protease cathepsin D in the cytosolic fraction of GNF-treated lung carcinoma cells [45]. Moreover, the reduced intracellular levels of lysosomal cathepsins in GO-treated monocytic leukemia cells indicate that GO nanoparticles might additionally affect lysosomal function by interfering with cathepsin expression [47]. Finally, GNF-triggered lysosomal dysfunction in lung carcinoma cells was associated with the disruption of actin cytoskeleton [45], which is consistent with the ability of lysosomal cathepsins to degrade actin and its binding partner and regulator myosin [71]. Keeping in mind the important role of the actin cytoskeleton in autophagosome formation and fusion with lysosomes [72], it is possible that the lysosome leakage-mediated disruption of actin-autophagy connection by GNM might ultimately impair even the early stages of autophagic response.

## 6. Structure–Activity Relationship of GNM as Autophagy Modulators

The chemical composition, size, shape, and surface charge of nanoparticles determine the biological outcome of their interaction with cells [54]. Therefore, an important question is how the physico–chemical characteristics of GNM influence their ability to modulate autophagy in cancer cells. One of the key issues in this respect concerns the apparently contradictory findings showing GNM-mediated autophagy induction in some, and block of its completion in other reports. While this, at least in some studies, was possibly due to insufficiently explored status of autophagic flux, the role of GNM morphology and chemical structure is a logical explanation that should not be overlooked.

The studies reviewed here employed a wide variety of GNM, differing in lateral size (few to several hundreds of nm), thickness (1 to several nm), and shape (oval GQD, irregularly shaped GO flakes and nanoribbons, pure graphene nano-cones). However, the effects of these parameters on the autophagy-modulating capacity of GNM were difficult to estimate due to the lack of direct intra-study comparisons and with the cell type specificity confounding the comparison between different studies. Nevertheless, the fact that GO and GNF shared the ability to block autophagic flux [44,45,46] indicates that this effect was not entirely due to the unique physico–chemical properties of GNF, which consist of stacked chemically non-modified graphene nano-cones. On the other hand, it is tempting to speculate that the size of GNM could determine their effect on autophagy. Namely, GNM that blocked autophagic flux, including GNF with the mean length of 25 µm and GO flakes with lateral size > 500 nm [44,45,46], were consistently larger than GO (mean diameter 10–450 nm) and GQD nanoparticles (mean diameter 3–60 nm) that apparently did not interfere with autophagy completion [29,30,31,32,33,34,35,39,40]. The only exception was the study showing autophagy induction by GO nanoribbons of few µm in length [37], but the possibility that the increase in LC3-II was due to its reduced autophagic degradation could not be excluded as no flux measurement was performed. Therefore, it is possible that large GNM particles with their sharp edges and rough surface could physically disrupt actin cytoskeleton and/or lysosomal membrane, thus preventing autophagosome-lysosome fusion and/or causing lysosomal leakage and subsequent decrease of their degradative capacity. Accordingly, although both large (≈300 nm) and small (≈90 nm) GO nanosheets were endocytosed to the same extent by cancer cells, the former were significantly more cytotoxic [73]. Molecular dynamics simulations revealed direct insertion and lipid extraction as two distinct modes of phospholipid bilayer damage by graphene and GO nanosheets, acting in concert as lipid extraction creates pockets that accommodate direct insertion [74]. One would expect that larger nanosheets should create more extensive cuts in cell membranes, as well as that lipid extraction should scale with the surface area of the interacting graphene [75], since in simulations both sides of graphene nanosheets became covered with adsorbed phospholipids [74]. While the above data support the crucial role of GNM particle size in determining their impact on lysosomal integrity and autophagy, the experiments directly comparing the capacity of GNM particles of different sizes to modulate autophagic flux would help to resolve this important issue.

The few studies that assessed the surface charge of autophagy-modulating GNM demonstrated that they were negatively charged (approx. 10–30 meV) in cell culture medium at physiological pH [37,44,45,46,47]. Since all GNM share the graphene core, their surface charge is mainly determined by different functional groups introduced during preparation. The crucial role of different surface functional groups on autophagy modulation by GNM was revealed in a study that examined the effects of amino-, carboxy-, or hydroxy-GQD on autophagic responses in human lung carcinoma cells [40]. Amino-GQD were more potent autophagy inducers than hydroxy-GQD, while carboxy-GQD failed to trigger autophagic response [40]. Although the surface charge values of the three GQD preparations were not reported, the following order of surface electric potential could be inferred from the studies of differently functionalized nanoparticles: amino-GQD > hydroxy-GQD > carboxy-GQD [76,77,78,79,80]. Therefore, it is tempting to speculate that the autophagy-inducing capacity of GQD correlated with their surface charge. A potential explanation could be that positively (or less negatively) charged surface of nanoparticles might facilitate their interaction with the negatively charged surface of plasma or endolysosomal membranes harboring the members of the autophagy-controlling mTORC1 signaling pathway [53]. Indeed, autophagy induction by amino-GQD was associated with the suppression of mTORC1 activator AKT, which was not observed upon treatment with less effective hydroxy- or non-effective carboxy-GQD [40].

Finally, having in mind that GQD endocytosis was apparently required for autophagy induction [35,41], it is conceivable that the autophagy-modulating capacity of GNM might depend on the extent of their cellular internalization. However, as no comparative analysis has been performed to correlate the ability of different GNM nanoparticles to enter the cancer cells with their capacity to modulate autophagy, this issue remains to be investigated.

## 7. The Role of Autophagy Modulation in GNM-Induced Cancer Cell Death

With the exception of few studies in which the cytotoxicity of GNM was not observed or assessed [31,34,36,37], GNM-mediated modulation of autophagy in tumor cells was consistently associated with the induction of apoptotic, necrotic, or necroptotic death [29,30,32,33,38,39,40,41,42,43,44,45,46,47]. Therefore, a question arises as to the role of autophagy modulation in GNM-induced death of cancer cells. Somewhat surprisingly, several studies that demonstrated the simultaneous occurrence of autophagy modulation and cytotoxicity did not directly examine a possible relation between the two events in GNM-exposed cancer cells [29,33,38,41,42,47]. The available data on this issue indicate that both autophagy induction and suppression of autophagic flux reduce cancer cell survival by promoting other types of cell death, as summarized in Table 2 and discussed below.

### 7.1. Cytotoxic Effect of GNM-Mediated Autophagy Induction

The suppression of autophagy induction by genetic inactivation of LC3 abrogated the cytotoxicity of photoexcited GQD towards human glioma cells, indicating a role for autophagic response in their photodynamic pro-apoptotic action [39]. GQD have been found to accumulate in the autophagolysosomal compartment [33,37,39], and photodynamic damage of lysosomes, as opposed to that of mitochondria, cannot be efficiently counteracted by autophagy [81]. Moreover, the release of calcium ions from photodamaged lysosomes activates the protease calpain, which then cleaves ATG5 to a mitochondria-binding pro-apoptotic fragment [81]. Therefore, it is conceivable that autophagy could contribute to photodynamic glioma cell death through the accumulation of GQD-containing lysosomes and their subsequent photodamage and pro-apoptotic cleavage of ATG5. Similarly, GO nanoparticles potentiated cisplatin-induced death of human ovarian carcinoma and cervical carcinoma cells through induction of autophagy, as revealed by cell protection afforded by pharmacological inhibitors of autophagy and genetic knockdown of autophagy activators ULK1 or ATG7 [30,32]. The observed cytotoxic effect apparently did not require the completion of autophagic flux and was mediated by nuclear import of LC3 and subsequent induction of necrotic cell death through an unidentified mechanism [30,32]. These data indicate that autophagic proteins such as LC3 and/or ATG5, rather than autophagic digestion itself, might be involved in GQD- and GO-mediated cancer cell death.

### 7.2. Cytotoxic Effect of GNM-Mediated Blockade of Autophagic Flux

GO-induced death of pheochromocytoma and glioma cells was apparently mediated by a blockade of autophagic flux [44,46]. Accordingly, restoring the autophagic flux with mTORC1 inhibitor rapamycin protected the cancer cells from GO-mediated apoptotic death [44,46]. GO triggered the autophagic response but blocked its completion by alkalizing the lysosomes, ultimately causing their dysfunction and subsequent accumulation of autophagic cargo receptor p62, which participated in the induction of caspase-dependent apoptosis [44,46]. In lung carcinoma cells, GO sheets conjugated with the lysosomal inhibitor chloroquine caused excessive accumulation of autophagosomes, with p62 and ATG5 serving as scaffolds for necrosome assembly and subsequent execution of necroptosis by necrosome components receptor-interacting protein kinases 1/3 and mixed lineage kinase domain-like protein [43]. Consequently, genetic inactivation of p62 rescued the cancer cells from GO/chloroquine-induced necroptotic death [43]. Similarly, GNF-mediated oxidative stress and apoptotic death of lung carcinoma cells were associated with impaired lysosomal function and increased accumulation of autophagosomes, LC3, and p62 [45]. Genetic inactivation of autophagy upon LC3 knockdown did not further augment, but rather reduced cell demise [45]. Collectively, these data indicate that the accumulation of autophagy mediators, rather than blocking the cytoprotective action of autophagy, was involved in GNM-triggered apoptotic or necroptotic death. However, it should be noted that the cytotoxicity of amino- and hydroxy-GQD in lung carcinoma cells was increased by 3-methyladenine [40], which suppresses autophagosome formation by blocking class III PI3K and its subsequent interaction with beclin-1 [82]. While suggesting a cytoprotective role of GQD-triggered autophagy, this needs to be confirmed through genetic approaches, particularly since 3-methyladenine exerts many autophagy inhibition-independent effects and could even induce autophagy through suppression of PI3K class I-mediated activation of AKT/mTORC1 [82]. Finally, both GO and GNF caused lysosomal membrane permeabilization [44,45], indicating a possible role of lysosomal proteases and subsequent ATG5 cleavage in their cytotoxicity.

## 8. Implications of Autophagy Modulation by GNM for Anticancer Therapy

The above-analyzed data indicate that accumulation of autophagy mediators resulting from the induction of autophagic response and further augmented by subsequent blockade of autophagic flux, might contribute to cancer cell killing by GNM. This indicates that the drugs that block autophagic degradation, such as clinically approved lysosomal inhibitors chloroquine and hydroxychloroquine [83], could improve the anticancer effects of GNM. However, to constitute a plausible anticancer strategy, this needs to be further analyzed, both functionally and mechanistically, as well as validated in the in vivo conditions. Moreover, there are other important concerns, such as the biocompatibility/biological fate of GNM and selectivity of GNM-mediated autophagy modulation and/or ensuing cytotoxicity. In addition, GNM are more likely to be used in cancer therapy as drug delivery systems or adjuncts to standard treatments, thus predicting the possible effects of GNM-mediated autophagy modulation in such settings would be of great importance. Finally, there is an issue of possible interference of GNM-modulated autophagy with anti-tumor immune response. These additional concerns are addressed in more detail below.

### 8.1. Biocompatibility and Biological Fate of GNM

In biodistribution studies in animals, intravenously injected GNM entered liver, spleen, lungs, and brain, and were efficiently excreted through urine and bile [84,85,86]. Moreover, GNM are degraded by human peroxidases [87,88], thus further reducing the risks of toxicity associated with prolonged accumulation in the tissues. While GQD were nontoxic, intravenous administration of GO/rGO in some studies caused lung inflammation, liver damage, and an increase in blood–brain barrier permeability, particularly at higher doses (≥5 mg/kg) [84]. In other studies, no significant toxicity was observed, which could be partly explained by different amounts of toxic residuals such as organic solvents, surfactants, strong acids, and oxidants used for exfoliating graphene flakes [84]. An important determinant of the biological fate and effects of nanoparticles is the formation of the so-called bio-corona, a coating of biomolecules (proteins, lipids, polysaccharides) that nanoparticles rapidly acquire from their surroundings in biological media [89]. Accordingly, experimental and theoretical approaches have demonstrated that protein coating can mitigate the cytotoxicity of GO by weakening the interaction between the membrane phospholipids and graphene surface, thus reducing graphene penetration and lipid bilayer damage [90]. On the other hand, an increase of ROS production has been observed in cancer cells exposed to plasma protein-coated graphene sheets [91]. Interestingly, it has been shown that the protein corona that forms around GO nanoparticles upon exposure to human plasma displays different compositions in healthy individuals and cancer patients, as well as in different types of cancer [92]. Therefore, it would be interesting to explore the effects of bio-corona, particularly the “personalized” protein corona originating from proteome alterations in different cancer types [93], on autophagy-modulating properties of GNM.

### 8.2. Selectivity of GNM-Mediated Autophagy Modulation in Anticancer Therapy

As with any anticancer therapy, it is important that autophagy modulation by GNM is selectively directed to cancer cells, thus increasing its efficiency while minimizing the toxicity towards healthy cells and tissues. Tumors can be selectively targeted by GNM through selective light exposure in photodynamic therapy, enhanced permeability and retention effect, and/or GNM surface functionalization with ligands for tumor-specific receptors [94]. Nevertheless, as it is still expected that a certain number of healthy cells will be exposed to GNM during the therapy, it is important to assess the responses of normal cells to autophagy-modulating action of GNM. While in some studies cytotoxicity was observed [95,96,97,98,99], GNM-induced autophagy in healthy, non-transformed primary cells was usually not associated with significant cell death [100,101,102,103,104,105,106]. The certain types of normal cells such as mouse lymphocytes and monkey fibroblasts were apparently resistant to both autophagy-modulating and cytotoxic action of GNM [107,108]. Interestingly, in a direct comparison under the same experimental conditions, GO/chloroquine conjugate at moderate concentration (25 µg/mL) induced autophagy-dependent necroptosis in lung carcinoma cells, but not in their non-cancerous counterparts [43]. While the apparently higher sensitivity of cancer cells to GNM-mediated autophagic death might be due to an increased volume and fragility of their lysosomes [64], this hypothesis remains to be experimentally tested. Nevertheless, fine-tuning of the physico–chemical properties of GNM to achieve selective autophagy-dependent cancer cell killing and better biocompatibility will be required for their further development as anticancer agents.

### 8.3. Autophagy Modulation by GNM as Chemo/Radio-Sensitizers and Drug Delivery Systems

As previously discussed, GO sensitized ovarian and colon carcinoma cells to cisplatin treatment through synergistic induction of autophagy and subsequent nuclear import of LC3, causing necroptotic cell death [30,32]. Similarly, the sensitization of cervical cancer cells to cisplatin-mediated apoptosis by rGO-silver nanocomposites was associated with synergistic expression of several ATG genes and subsequent accumulation of autophagic vesicles [109]. GO nanosheets with FePt nanoparticles deposited on their surface increased the sensitivity of lung carcinoma cells to X-ray radiation both in vitro and in vivo, potentiating its effects on autophagosome accumulation and induction of autophagic flux [110]. However, the role of autophagy in the chemo/radio-sensitizing effect of rGO-silver and GO-FePt nanocomposites was not directly confirmed. Photothermal therapy with GO loaded with heat shock protein 70 inhibitor and folic acid for tumor-selective delivery induced AKT inhibition-dependent autophagy in osteosarcoma xenografts [111]. While the in vitro experiments indicated a cytoprotective role of autophagy in this setting, this possibility was not assessed in vivo. A multifunctional nanoplatform for the simultaneous delivery of doxorubicin and the inhibitor of DNA-repairing protease MutT homolog 1 was designed using GO modified with folic acid, polyethylene glycol, and indocyanine green to achieve tumor selectivity, reduced immunogenicity, and photodynamic cancer cell killing, respectively [112]. The chemo-photodynamic therapy with this nanoassembly was efficient against osteosarcoma xenografts in vivo and caused ROS-mediated increase in beclin-1 expression and autophagic flux in osteosarcoma cells in vitro [112]. However, it remains to be assessed whether GO, besides serving as a drug delivery scaffold, could directly contribute to the observed anticancer effect by inducing autophagy.

### 8.4. Immunomodulatory Effects of GNM-Induced Autophagy

Accumulating evidence reveals an important, dichotomous role of autophagy at the tumor-immune interface. While participating in antigen processing and presentation, which are essential for T cell-dependent anti-tumor immune responses, autophagy can also reduce immunogenicity of tumors and promote their resistance to the cytotoxic effects of natural killer cells, macrophages, and T lymphocytes [113]. We have found that GQD-mediated autophagy induction in dendritic cells was associated with the decrease in their capacity to induce anti-tumor cytotoxicity of tumor antigen-primed T cells [101]. Moreover, instead of supporting the proliferation and development of pro-inflammatory helper T cells, GQD-exposed dendritic cells triggered the expansion of immunosuppressive regulatory T lymphocytes. Genetic silencing of ATG5, beclin-1, or p62 impaired the ability of GQD to induce tolerogenic dendritic cells, indicating the involvement of autophagy in this process [101]. On the other hand, photothermal therapy of osteosarcoma xenografts with heat shock protein 70 inhibitor/folic acid-loaded GO increased T cell-mediated anti-tumor immune response [111]. While autophagy participated in the ability of GO-based nanocomposite to relieve the T cell response from programmed death ligand-1 suppression in vitro, it was not assessed if such a mechanism might be operable in vivo [111]. Together, these data indicate a context-dependent dual role of GNM-induced autophagy in the modulation of anti-tumor immune response.

## 9. Conclusions and Future Directions

**Figure 4:**
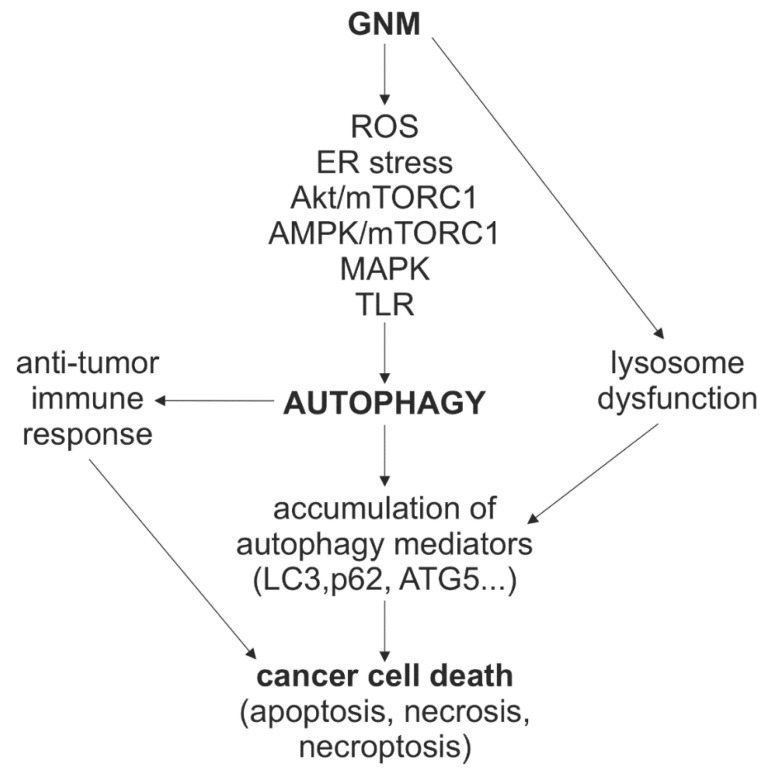
A schematic representation of the mechanisms and role of autophagy modulation in the anticancer action of GNM. GNM induce autophagy through oxidative/ER stress and AMPK/AKT/mTORC1, MAPK, or TLR signaling, but can simultaneously block autophagic flux by causing lysosomal dysfunction. This leads to accumulation of autophagic mediators such as LC3, p62, and ATG5, which participate in apoptotic, necrotic, or necroptotic death of tumor cells. GNM-induced autophagy could also modulate anti-tumor immune responses. AMPK, AMP-activated protein kinase; ATG, autophagy-related; ER, endoplasmic reticulum; GNM, graphene nanomaterials; LC3, microtubule-associated light chain 3; MAPK, mitogen-activated protein kinases; mTOR, mechanistic target of rapamycin complex 1; ROS, reactive oxygen species; TLR, Toll-like receptors.

Despite the fact that theoretical and experimental exploration of graphene had already commenced in the 20th century [114,115], and that it was unambiguously produced and identified more than 15 years ago [116], the research on biomedical applications of GNM is still in its early phase. Although many exciting medical uses are envisaged, the standardized procedures for large-scale preparation of impurity-free GNM with well-defined physico–chemical features and satisfactory biocompatibility profiles are required before possibly embarking on clinical trials [117]. Nevertheless, basic research of GNM application in medicine has been on the rise in the last decade, with autophagy modulation-based anticancer therapy emerging as one of its most recent advances. The anticancer effect of GNM is at least partly mediated by the induction of autophagy and/or the suppression of autophagic flux, leading to the accumulation of autophagic mediators that participate in apoptotic, necrotic, or necroptotic death of tumor cells, as well as in the modulation of anti-tumor immune responses (Figure 4). GNM activated autophagy through oxidative/ER stress and AMPK/AKT/mTORC1, MAPK, or TLR signaling, while the blockade autophagic flux was apparently associated with the ability of large GNM nanoparticles to cause lysosomal dysfunction (Figure 4). Collectively, this indicates that the inhibition of late, rather than early stages of autophagy could be a feasible approach to improve the anticancer performance of GNM. However, a deeper understanding of GNM-autophagy interaction at the mechanistic and functional level is needed before these findings could be exploited to increase GNM effectiveness as cancer therapeutics and drug delivery systems. The crucial aspects in this regard are how therapeutic modality (e.g., photodynamic vs. photothermal therapy vs. no photoexcitation) and physico–chemical properties of GNM influence their ability to modulate autophagy and kill cancer cells directly or in conjunction with other agents. Answering these questions will require comparative analysis of structure–activity relationships and combination therapies, genetic and omics approaches to discern the molecular mechanisms and biological consequences of GNM-mediated autophagy modulation, as well as in vivo experimental models to address its therapeutic feasibility.

## Figures and Tables

**Table 1:** GNM-mediated autophagy modulation in cancer cells.

GNM	Cell Type	LC3-II, LC3 Puncta	AV (TEM)	Autophagic Flux	Mechanisms	Ref.
GO	MG-63 human osteosarcoma	↑	n.a.	↑ (↑LC3-II—flux assay)	↑ROS, ↑ATG3, ↑ATG5, ↑ATG7, ↑NRF2, ↓BCL2	[29]
GO	CT26 mouse colon carcinoma, Skov-3 human ovarian carcinoma	↑	n.a.	↑ (AP-LY fusion)	n.a.	[30]
GO	SH-SY5Y human neuroblastoma	↑	n.a.	↑ (↓p62)	↑AMPK, ↓mTOR, ↑beclin	[31]
GO	CT26 mouse colon carcinoma	↑	n.a.	↑ (AP-LY fusion)	n.a.	[32]
GO	CT26 mouse colon carcinoma	↑	↑	↑ (AP-LY fusion)	↑TLR4/TLR9, ↑MyD88, ↑TRAF6, ↑NF-κB, ↑beclin	[33]
GO	SK-N-SH human neuroblastoma	↑	↑	↑ (↓p62)	n.a.	[34]
GO	PC12 rat pheochromocytoma, HeLa cervical carcinoma	↑	↑	↑ (↑LC3-II—flux assay)	↑ERK	[35]
GO	RAW264.7 mouse monocytic leukemia	↑	↑	n.a.	↑TLR4/TLR9, ↑MyD88, ↑TRAF6, ↑NF-κB, ↑beclin	[36]
GO	SK-N-BE(2) and SH-SY5Y human neuroblastoma	↑	↑	n.a.	↑ROS, ↑BNIP3, ↑beclin	[37]
GO	HONE1 human nasopharingeal carcinoma	↑	↑	n.a.	↑LC3 mRNA, ↑ER stress, ↑GRP78	[38]
blue light-excited GQD	U251 human glioma	↑	↑	↑ (↓p62)	↑ROS	[39]
hydroxy-GQD	A549 human lung carcinoma	↑	↑	? (→p62)	↑p38MAPK, ↑JNK	[40]
amino-GQD	A549 human lung carcinoma	↑	↑	? (→p62)	↑p38MAPK, ↓Akt, ↑beclin	[40]
GQD	THP-1 human monocytic leukemia	↑	n.a.	n.a.	↑ROS, ↑p38MAPK, ↑NF-κB, ↑beclin, ↓BCL2	[41]
GO, GO-Ag composite	SH-SY5Y human neuroblastoma	n.a.	↑	n.a.	↑ROS, ↓BCL2 mRNA	[42]
GO-CQ conjugate	A549 human lung carcinoma	↑	↑	↓ (↑p62)	↑ROS, ↑ATG5	[43]
GO	PC12 rat pheochromocytoma	↑	↑	↓ (↓AP-LY fusion, ↑p62)	↓PI3K, ↓Akt, ↓mTOR, ↑ATG5, ↓BCL2, ↓LY acidity, ↓ACP, ↓CTSB, ↑LMP	[44]
GNF	A549 human lung adenocarcinoma	↑	↑	↓ (↑p62)	↑ROS, ↓mTOR, ↑ATG5, ↓BCL2, ↑beclin, ↑beclin/LC3/p62 mRNA, ↓LY acidity, ↑LMP, ↓actin cytoskeleton	[45]
GO	F98 rat glioma	↑	↑	↓ (↑p62)	↓PI3K, ↓Akt, ↓mTOR, ↓LY acidity, ↓CTSB	[46]
GO	RAW264.7 mouse monocytic leukemia	↑	n.a.	↓ (↑p62)	↑ROS, ↑NRF2, ↓CTSB, ↓CTSD	[47]

↑, increase/activation; ↓, decrease/inhibition; →, no change; ?, cannot be estimated based on the available data; n.a., not assessed; ACP, acid phosphatase; AMPK, AMP-activated protein kinase; AP, autophagosome; ATG, autophagy-related; AV, autophagic vesicles; BCL2, B-cell lymphoma 2; BNIP3, BCL2-interacting protein 3; CTS, cathepsin; CQ, chloroquine; ER, endoplasmic reticulum; GNF, graphene nanofibers; GNM, graphene nanomaterial; GO, graphene oxide; GQD, graphene quantum dots; GRP78, glucose-regulated protein 78; JNK, c-Jun N-terminal kinase; LC3, microtubule-associated light chain 3; LMP, lysosome membrane permeabilization; LY, lysosome; MAPK, mitogen-activated protein kinase; mTOR, mechanistic target of rapamycin; MyD88, myeloid differentiation factor 88; NF-κB, nuclear factor-κB; NRF2, nuclear factor E2-related factor 2; PI3K, phosphoinositide 3 kinase; ROS, reactive oxygen species; TEM, transmission electron microscopy; TLR, Toll-like receptor; TRAF6, TNF receptor-associated factor 6.

**Table 2:** The role of autophagy modulation in GNM-induced cancer cell death.

GNM	Cell Type	Type of Cell Death	Autophagy Involvement	Cell Death Modulation	Cell Death Mechanisms	Ref.
GO + cisplatin	Skov-3 human ovarian carcinoma	necrosis	cytotoxic autophagy induction	↓ by ULK1 RNAi ↓ by ATG7 RNAi	nuclear import of LC3	[30]
GO + cisplatin	CT26 mouse colon carcinoma	necrosis	cytotoxic autophagy induction	↓ by 3-MA ↓ by BAF A1	nuclear import of LC3	[32]
blue light-excited GQD	U251 human glioma	apoptosis	cytotoxic autophagy induction	↓ by LC3B RNAi	↑ROS, ↑caspases	[39]
hydroxy-GQD	A549 human lung carcinoma	apoptosis	cytoprotective autophagy induction	↑ by 3-MA	n.a.	[40]
amino-GQD	A549 human lung carcinoma	apoptosis upon autophagy inhibition	cytoprotective autophagy induction	↑ by 3-MA	n.a.	[40]
GO-CQ conjugate	A549 human lung carcinoma	necroptosis	cytotoxic block of autophagic flux	↓ by p62 RNAi	↑ROS, ↑RIPK1, ↑RIPK3, ↑MLKL, p62 and ATG5 as scaffolds for necrosome assembly	[43]
GO	PC12 rat pheochromocytoma	apoptosis	cytotoxic block of autophagic flux	↓ by rapamycin ↓ by p62 RNAi	↑p62, ↑LMP, ↓ΔΨ, ↑Bax/Bcl-2, ↑caspase-3, ↑caspase-9	[44]
GNF	A549 human lung carcinoma	apoptosis	cytotoxic block of autophagic flux	↓ by 3-MA ↓ by LC3 RNAi	↑ROS, ↑LMP, ↓ΔΨ, ↑Bax/Bcl-2, ↓ATP, ↑caspase-3, ↓actin cytoskeleton	[45]
GO	F98 rat glioma	apoptosis	cytotoxic block of autophagic flux	↓ by rapamycin	↑p62, ↑Bax/Bcl-2, ↓ΔΨ, ↑caspase-3	[46]

↑, increase/activation; ↓, decrease/inhibition; n.a., not assessed; ΔΨ, mitochondrial membrane potential; 3-MA, 3-methyladenine; ATG, autophagy-related; BAF, bafilomycin; CQ, chloroquine; CTS, cathepsin; GNF, graphene nanofibers; GO, graphene oxide; GQD, graphene quantum dots; LC3, microtubule-associated light chain 3; LMP, lysosome membrane permeabilization; MLKL, mixed lineage kinase domain-like; RIPK, receptor-interacting protein kinase; ROS, reactive oxygen species; RNAi, RNA interference; ULK, Unc-51-like autophagy activating kinase.
